# The potential of statistical shape modelling for geometric morphometric analysis of human teeth in archaeological research

**DOI:** 10.1371/journal.pone.0186754

**Published:** 2017-12-07

**Authors:** Christopher Woods, Christianne Fernee, Martin Browne, Sonia Zakrzewski, Alexander Dickinson

**Affiliations:** 1 Bioengineering Sciences Research Group, University of Southampton, Highfield Campus, Highfield, Southampton, United Kingdom; 2 Department of Archaeology, University of Southampton, Avenue Campus, Highfield, Southampton, United Kingdom; Monash University, AUSTRALIA

## Abstract

This paper introduces statistical shape modelling (SSM) for use in osteoarchaeology research. SSM is a full field, multi-material analytical technique, and is presented as a supplementary geometric morphometric (GM) tool. Lower mandibular canines from two archaeological populations and one modern population were sampled, digitised using micro-CT, aligned, registered to a baseline and statistically modelled using principal component analysis (PCA). Sample material properties were incorporated as a binary enamel/dentin parameter. Results were assessed qualitatively and quantitatively using anatomical landmarks. Finally, the technique’s application was demonstrated for inter-sample comparison through analysis of the principal component (PC) weights. It was found that SSM could provide high detail qualitative and quantitative insight with respect to archaeological inter- and intra-sample variability. This technique has value for archaeological, biomechanical and forensic applications including identification, finite element analysis (FEA) and reconstruction from partial datasets.

## Introduction

Anthropometric analysis is an essential area of research in understanding development, health and disease, and informs a range of work notably including medicine, biomedical engineering, forensics, and archaeology. Archaeologically, teeth are studied because they are “made up of the two hardest tissues in the body [and] so are … most likely to be preserved in the fossil and archaeological records.”[1]. The study of human dental morphology permits the reconstruction of diet, past population migration patterns and the understanding of health and cultural practices. Given the nature of the archaeological record, and its fragmentary and punctuated character, the question arises as how to actually analyse such complex forms.

**Fig 1:**
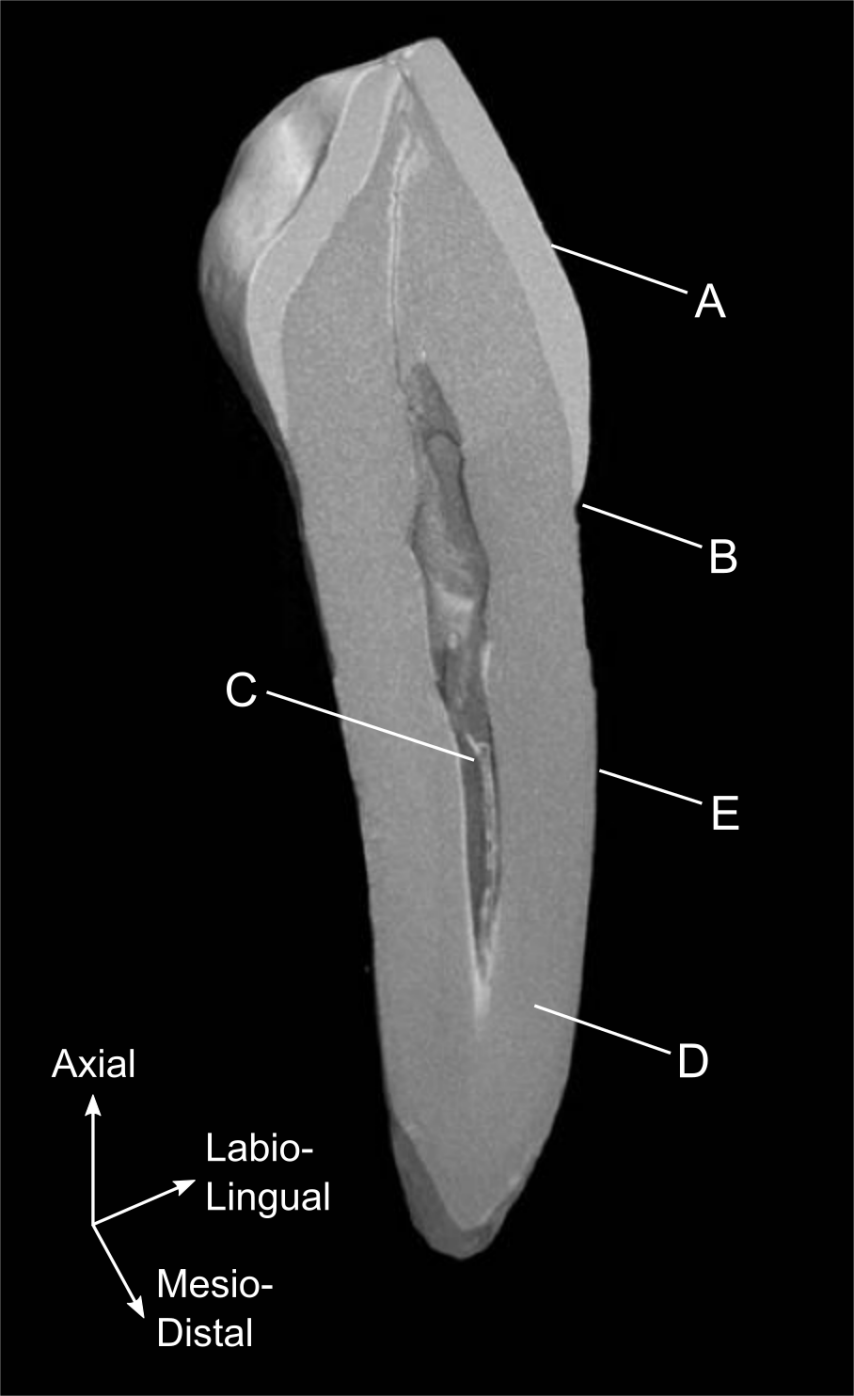
Canine CT. Canine tooth section obtained from micro-CT. (A) Enamel crown, (B) cemento-enamel junction, (C) pulp chamber, (D) dentin root and (E) cementum layer.

Archaeological study of teeth for the investigation of human variation relies on a morphological understanding of the root (cementum coated), crown (enamel coated) and their interface the cemento-enamel junction (CEJ) (Fig 1). Analysis of dental morphology is either metric or non-metric [2]. Non-metric studies of dental morphology have analysed the presence, absence or the degree of expression of particular quasi-continuous features in both archaeological and modern samples [3, 4]. Non-metric studies are used to identify population history, migration patterns and differences between population groupings, as it has been suggested that qualitative characteristics are more useful in grouping people according to their locations and affinities [5–8]. Metric analysis, at its very simplest, consists of gross measurement of the tooth in two planes, traditionally the mesiodisal and labiolingual crown diameter [9–12] and more recently, around the CEJ [13]. Advancements in imaging techniques have progressed the field of metric dental analysis as measurements can now be taken in two and three dimensions [14] with a high degree of accuracy [5].

Morphometrics is the term used when multivariate statistical techniques are applied to linear metric measurements to make comparisons between samples [15–18]. The limitations of traditional morphometrics, such as interrelationships and covariation between variables, have given rise to geometric morphometrics (GM), whereby landmarks and semi-landmarks are manually or semi-automatically mapped on an object to enable the analysis of outlines and complex surface geometries [19, 20]. GM evaluates shape variation and its covariation with other variables, and thus has been used to describe morphological differences both within and between populations [17, 18, 21, 22].

Dental studies that adopt GM fall into three main categories: taxonomy, ecology and adaptation. Of these the most prevalent are studies of taxonomy, proving useful in studying hominoid evolution and identifying taxonomic variation [23–29]. The majority of these studies employed outline or surface methods to compare occlusal morphology, either in 2D or 3D. These have primarily been applied to hominin dental samples to analyse variation of crown morphology, CEJ or dentin-enamel morphology, and thereby distinguish between taxa [30–34] or describe characteristics of specific hominin fossil specimens [35–37]. Occasionally, tooth tissue proportions have been studied alongside GM analysis of the CEJ, although each data type has been considered separately [38–41]. Rarely do studies consider tooth surface, volume and area in combination. GM analysis has been used to quantify anterior tooth root shape in hominids alongside independent variables including linear, surface area and volumetric measurements [42].

To date, dental GM studies have rarely focussed on modern humans (notable exceptions include the work by Polychronis and colleagues) [43, 44] or recent archaeological specimens (unless as comparator samples) [41]. It is applied occasionally to modern samples in a clinical setting to study dental anomalies [45], malocclusion and modern dental variation [43]. We have recently reported on the state of the art for inter-population GM analysis of modern and archaeological teeth [46].

Statistical Shape Modelling (SSM) is a supplementary morphometric technique akin to GM, which assigns ‘landmarks’ to an object’s surface. SSM exploits recent improvements in imaging resolution to assign a very high density of landmarks to an object’s surface such that full-field geometric variations can be represented statistically. Using SSM it is possible to make both metric and non-metric observations of the dominant variations within a sample [47]. This paper thus presents the first application of SSM to archaeological remains.

GM enables the morphology of an entire tooth to be used as the framework for statistical analysis. This is important given the lack of identifying landmarks in portions of the tooth structure, such as over the human tooth root. GM however does not provide information as to the material composition. By contrast, SSM enables complex shape structure and material properties to be analysed together. The resulting data can then be imported into other packages for further statistical analysis. The approach thus has major potential for archaeology and anthropology given the paucity of complete objects, items or collections for study. By synthesising material properties with shape, more information may be extracted from each sample or object analysed. This is especially valuable with archaeological materials, where, by their very nature, the sample size is liable to be finite and small.

Statistical shape models reduce the dimensionality of a training database of geometries into linearly uncorrelated parameters which can be used to describe geometric variation across a population. This is achieved using PCA which decomposes the training population into a series of orthogonal matrices [47, 48]. Statistical models are used in biological applications due to their ability to capture complex anatomical geometric variation, and thus SSM has been applied to both soft tissues [49, 50] and hard tissues [51, 52]. By manipulating the model, it is possible to create unique instances of synthetic geometry whose variation is ‘legal’ within the training population. When the training data is taken from x-ray CT (computer tomography) imaging, it is possible to include a greyscale intensity parameter to create a statistical shape and intensity model (SSIM) [53]. In orthopaedic applications, by correlating intensity directly to density and thus to material parameters such as the elastic modulus, it is possible to create a synthetic geometry including both material properties and geometric information [54, 55].

SSM has two distinct strengths within the context of other GM methods; data resolution and incorporation of material.

SSM, as a method, represents an object at its highest available resolution (determined by scanning protocol). Data down-sampling is only performed when necessary to minimise computational cost. This contrasts with landmark based GM where data resolution is determined through the selection of landmarks and then increased with the assignation of semi-landmarks. The cost of high resolution data mapping is a loss of homologous data points. However, this may be an advantage in applications such as anatomy, where landmarks can be ambiguous due to the continuous nature of the geometry. Similarly, forcing landmarks in GM may bias models towards major anatomical features whist obfuscating geometric subtleties. As these methods are fully automated, they are not subject to the inter/intra-observer errors attributed to the experience level of the operator at assigning landmarks.

Including material information is an advantage of SSM that can further benefit GM methods. To the authors knowledge, materials have not supplemented geometry in archaeological GM analysis to date. In many applications changes in material represent functional attributes. By modelling material alongside geometry, it is possible to delineate objects or assign landmarks post hoc. This serves to increase the analytical possibilities on any heterogeneous structure, be it osseous, lithic or ceramic.

The current study aimed to demonstrate the use of the statistical shape analysis approach for archaeological samples. It employs micro-CT imaging to construct a SSM of human canine dental samples. The model uses surface geometry and material information such that the CEJ can be identified and measured as a material rather than a geometric transition, as it is in reality. The model is used to characterise qualitatively and quantitatively the principal modes of variation within the dataset, before demonstrating how these variations might be used to identify differences between populations. The method could be extended to samples currently starting to be analysed using GM approaches, such as ceramic and lithic artefacts.

## Methods

### Data acquisition

#### Collection

Three adult human mandibular canine populations were sampled. Canines were selected as, following Butler’s field theory, they are considered the most stable tooth within the dentition, exhibiting the least variation [56, 57]. The first sample (n = 9) was a modern human (**MH**) dataset. These were collected from routine extraction procedures (Essex, UK) in accordance with a local (Ethics and Research Governance Online, Ref. 6870) and national (National Research Ethics Service, Ref 12.LO.0901) ethics committee approved protocol. The second sample (n = 13) was collected from Great Chesterford (**GC**), an Anglo-Saxon burial site (Essex, UK) circa 5^th^ - 7^th^ century AD. The final sample (n = 5) was collected from Huntsman’s Quarry (**HQ**), a Roman (Worcestershire, UK) burial site circa 2^nd^ - 3^rd^ century AD. Inclusion criteria required the teeth to be free from damage, significant wear (Molnar score < 6 [58]) or gross malformation. A total of 27 samples met the inclusion criteria, and are referred to as the ‘training data’.

#### Digitisation

The teeth were scanned using a Custom 225kV Nikon/Metris HMX ST micro-CT scanner at 110 kV and 165 *μ*A, for a target resolution of 30 μm. The images were reconstructed using CT Pro software (Nikon Metrology, Herts, UK), resulting in a voxel size of 27.8 x 30.4 x 30.4μm. The data were segmented using ScanIP (Simpleware, Exeter, UK) based on thresholding criteria to create individual masks for enamel, dentin and pulp chamber. For the purposes of this study, cementum was included in the dentine mask as only the external tooth geometry was required. For each threshold range a surface mesh was generated and exported, resulting in three unique meshes for each tooth: enamel, dentin and whole tooth (enamel & dentin combined). A whole tooth surface mesh consisted of approximately 30k nodes.

### Registration

Landmarks are used to indicate the correspondence between points on a series of geometries in a point distribution model (PDM). Landmarks can be applied manually, to distinguishable locations on a shape [59], or automatically [60]. For this application, automated mesh-to-mesh registration was used, whereby each node of the tooth surface mesh forms a landmark. This method of registration morphs a baseline mesh onto the target meshes of the training data, such that each tooth geometry is represented using the same number of nodes and elements. It is possible to reduce the dimensionality of the data by removing size, using Procrustes analysis [61], that often confounds shape. However, in this introduction to SSM, size and shape will be considered together in order to demonstrate the fundamental effects of SSM.

#### Alignment

The baseline mesh was scaled to the target mesh prior to the alignment process. Maximum and minimum measurements of the target were taken in the x, y & z directions, and the baseline was scaled accordingly. The target meshes were aligned with the baseline using a combination of origin alignment (translation), coarse manual alignment (user-defined rotation and translation increments) and more precise iterative closest point (ICP) matching algorithms, and the transformation matrices stored. The entire alignment procedure was implemented using a custom MATLAB (MathWorks, MA, USA) GUI (S1 Fig). This alignment procedure was used to reduce statistical noise caused by misalignment of the training data.

#### Elastic matching

Registration was achieved using an elastic matching algorithm [62] implemented with MATLAB. The algorithm was adapted to improve overall mesh quality and efficiency [55]. Quality was improved using Laplacian smoothing [63] within each iteration, and efficiency was improved using k-d trees to find nearest neighbours. Detailed implementation information can be found in Bryan and colleagues [64]. Once registered, the whole-tooth meshes were represented by the column vector:

$$x={\left[x_{1},y_{1},z_{1},\ldots ,x_{M},y_{M},z_{M}\right]}^{T}$$

where *M* is the number of nodes in the baseline mesh and *x*, *y & z* are the nodal coordinates.

The registration quality was subsequently improved by extracting the mean tooth geometry and using this as the baseline to then repeat the registration process for the training data [48]. The vector describing the mean baseline tooth, $\bar{x}$, was determined by:

$$\bar{x}=\frac{1}{N}\sum_{i=1}^{N}x_{i}$$

where *N* is the number of samples in the dataset. Each tooth was thus described by 26,795 nodal landmarks.

#### Enamel matching

The transformed enamel mesh was used to identify the surface material type on the registered whole tooth mesh. Euclidean k-d trees were constructed to locate the enamel nodes on the registered whole-tooth meshes. Therefore, each whole-tooth mesh was described by:

$$x={\left[x_{1},y_{1},z_{1},I_{1}\ldots ,x_{M},y_{M},z_{M},I_{M}\right]}^{T}$$

where *I* is a binary material identifier for enamel (*I* = 1) or dentin (*I* = 0).

### Statistical modelling

PCA was conducted using MATLAB on the registered, material identified, dataset. The correlation method was used due to the mixed geometry and material units [65]. Thus, each tooth could be described by:

$$x=\bar{x}+\sum_{j=1}^{c}\varphi_{j}d_{j}$$

where *φ*_*j*_ are the eigenvectors corresponding to the PCs and *d*_*j*_ is a vector containing the weighting coefficients associated with the eigenvectors. Using this relationship, legal or permissible synthetic geometries are created by defining different eigenvector weighting coefficients. For example, the mean tooth can be generated using *d*_*j*_ *= 0*. The number of PCs to include, *c*, is determined by the cumulative variance desired.

To illustrate the independent shape variation from the mean described by each PC, synthetic geometries were generated. These geometries were plotted as integer standard deviations (*σ*) from -3*σ* to +3*σ*, to represent the variation within the data.

### CEJ identification

**Fig 2:**
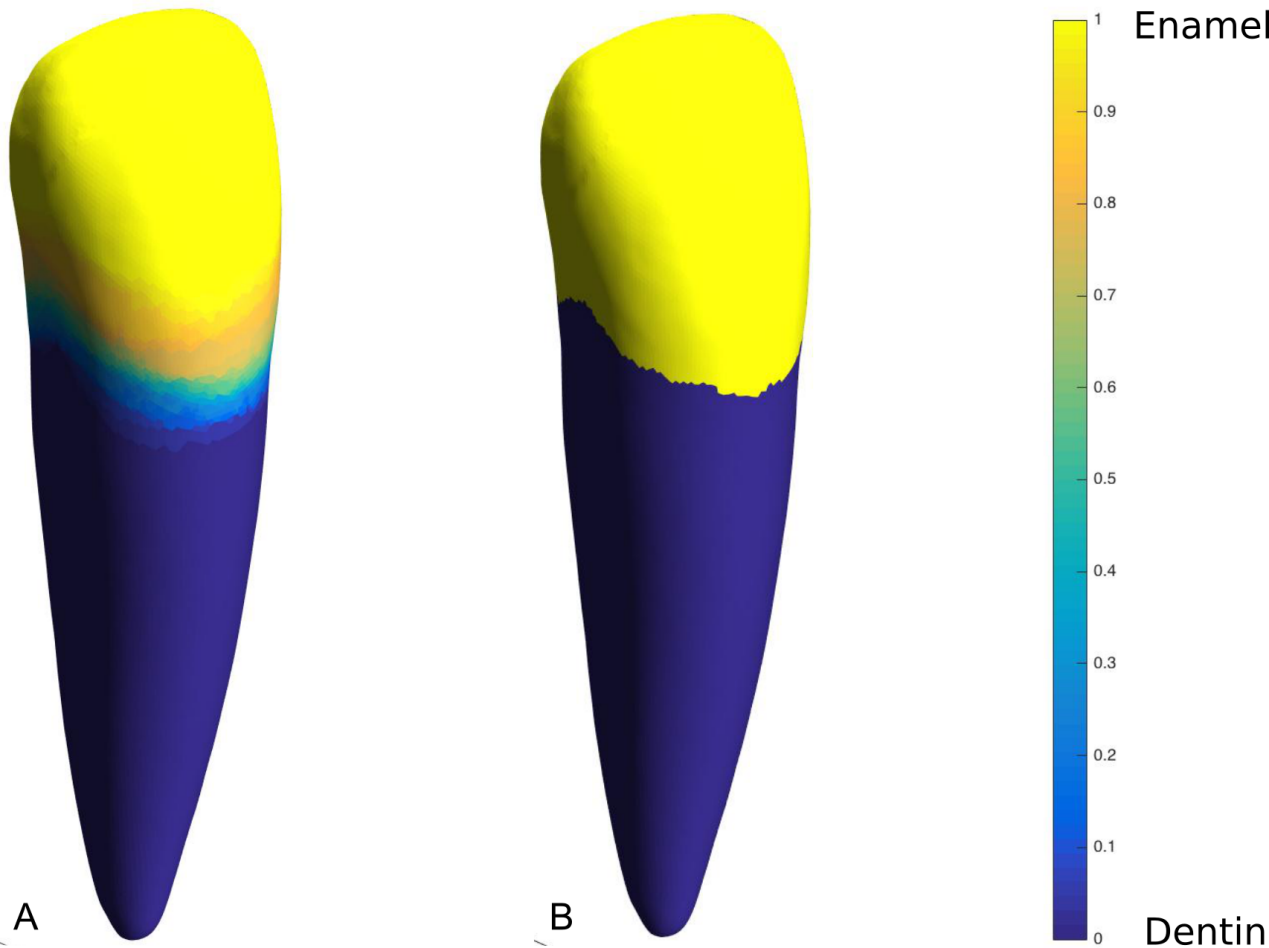
CEJ identification. CEJ identification: (A) Original data, (B) thresholded and smoothed data.

The surface material information of a synthetic geometry generated from SSM was probabilistic, ranging from *I* = 0 to 1. Therefore, it was necessary to identify an absolute, binarised CEJ boundary by thresholding above and below the mid-range value (Fig 2):

$$if\;I\geq 0.5\;then\;I_{h}=1$$

$${if\;I<0.5\;then\;I}_{h}=0$$

The anatomical demarcation of the CEJ is smooth, continuous and does not form isolated regions of cementum or enamel [66]. Measures were taken therefore to ensure that the synthetic geometries formed unbroken CEJ profiles. Noise at the CEJ was automatically smoothed in MATLAB using a custom script that identified the material (enamel or dentin) of elements bordering each element. Smoothing was achieved by changing the material identification of an element if two or more of its borders adjoined with elements of a different material type (S2 Fig).

### Synthetic geometry measurement

**Fig 3:**
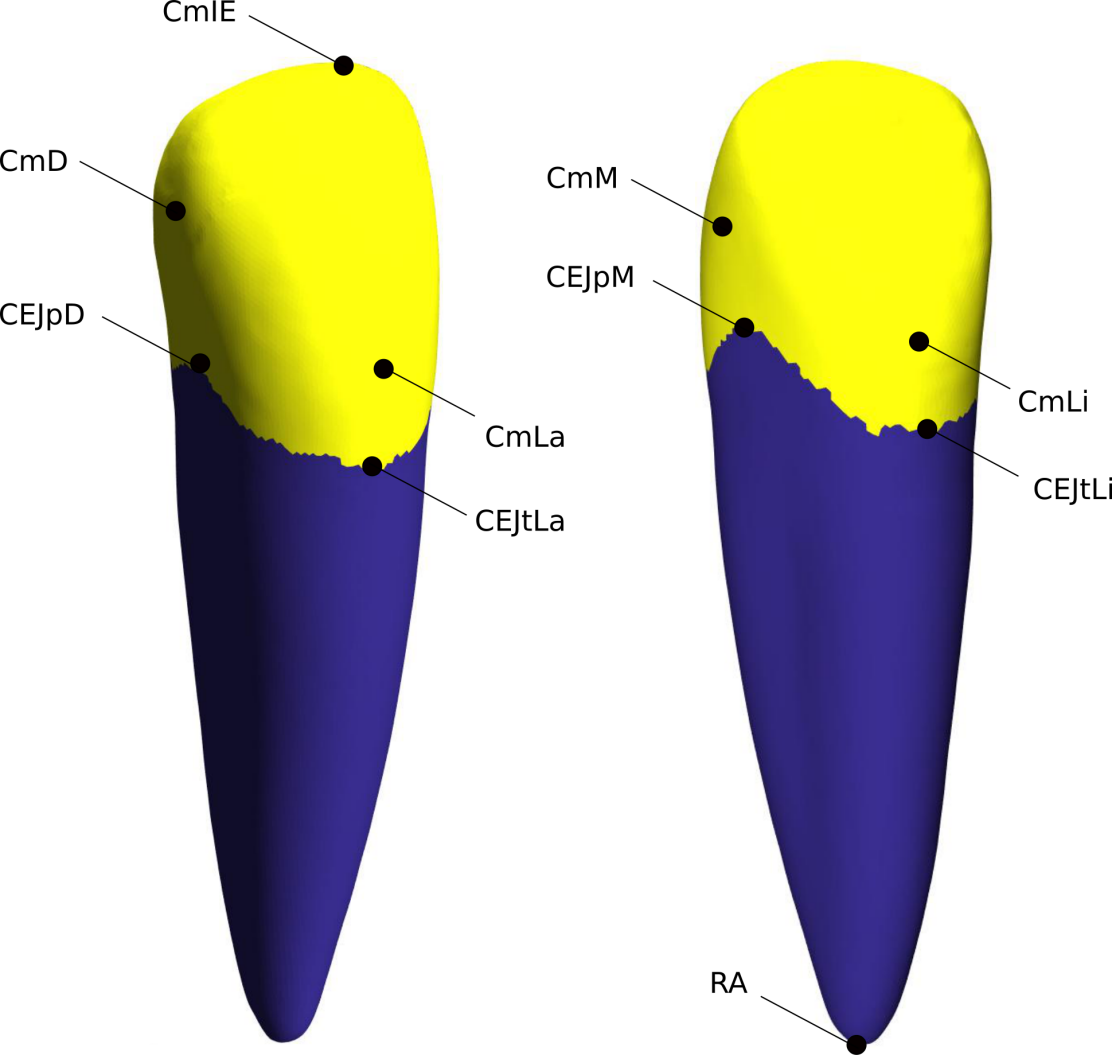
Tooth landmarks. Canine tooth with automatic landmark locations identified. Labio-distal view (left) and Lingual-mesial view (right).

Each tooth’s volume and surface area were calculated using in-built MATLAB algorithms. Key geometric landmarks (Table 1) were identified on the tooth surface, and gross measurements were extracted as the inter-landmark distances (Table 2). It was possible to use a local coordinate system to identify the landmarks as all synthetic geometry have a common alignment. Therefore, with knowledge of material type, and by partitioning each tooth into halves along the mesio-distal and labio-lingual planes, landmarks could be identified using maximum and minimum geometric criteria (Fig 3):

**Table 1:** Automated landmarks.

Landmark	Notation
Root apex	RA
CEJ trough–Labial Aspect	CEJtLa
CEJ trough–Lingual Aspect	CEJtLi
CEJ peak–Mesial Aspect	CEJpM
CEJ peak–Distal Aspect	CEJpD
Crown max–Mesial	CmM
Crown max–Distal	CmD
Crown max–Labial	CmLa
Crown max–Lingual	CmLi
Crown max–Incisal Edge	CmIE

**Table 2:** Automated measurements.

Measurement	Notation	Calculation
Labiolingual diameter of crown at cervix	LDCc	CEJtLa—CEJtLi
Labiolingual diameter of crown	LDC	CmLa—CmLi
Mesiodistal diameter of crown at cervix	MDCc	CEJpLa—CEJpLi
Mesiodistal diameter of crown	MDC	CmM—CmD
Length of root–labial	LORla	RA—CEJtLa
Length of crown–labial	LOCla	CEJtLa—CmIE
Volume	vol	Subroutine
Area	area	Subroutine

### Extracted measures

#### Reconstruction error test

The model was used to recreate the geometry of each tooth from the training data set using an increasing number of PCs and their known weighting coefficients. The error was calculated from the Euclidean distances for each point on the synthetic surface to the nearest point on the target surface found using a nearest neighbour search. The mean error was recorded for each test instance.

#### Automated measurements

A comparison of computational and manual measurements was performed to evaluate the reliability of the probed measurements (Table 2) of the training dataset. The Bland-Altman method [67], was used to assess bias, outliers, and changes in variance with measurement size [68].

#### Principal component analysis

The variation of canine geometry contained within each PC was assessed by two approaches. The first was a qualitative approach whereby the shape variation across each PC was inspected and described. The second method was a quantitative representation of the shape variation associated with each PC that compared the change in anatomic measurements with respect to the mean shape. This allowed for trends associated with each PC to be identified through relative changes in geometry and subsequent statistical analysis. A regression analysis was conducted for each automated measurement against PC weighting coefficient. A linear fit was mapped to each measurement series and the *R*^*2*^ values were recorded. Significance was assessed using p-Values (*α* = 0.01) from a linear regression t-test.

#### Sample characterisation

For each PC the weighting coefficients were assessed for statistically significant differences between the samples. A null hypothesis was constructed stating that all mean weighting coefficients were equal. One-way analysis of variance (ANOVA) tests were used first to identify differences in the mean weighting coefficients of the samples, and then, if a significant difference was identified, a post-hoc multiple comparison test was conducted to determine between which samples these differences were found.

## Results

### Statistical verification

**Fig 4:**
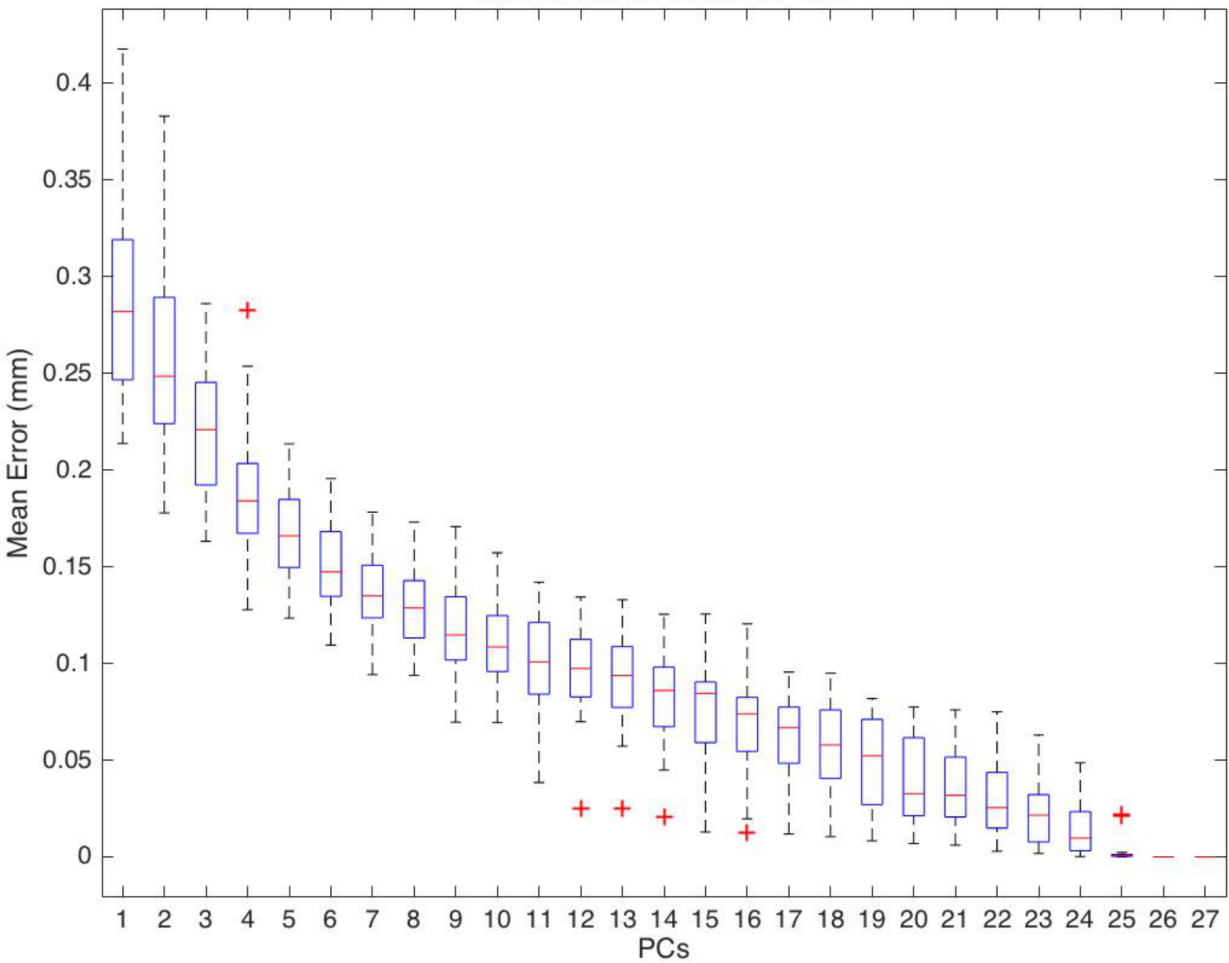
Reconstruction error. Mean surface reconstruction error boxplot for complete canine statistical model.

The robustness of the statistical model was assessed using a geometric reconstruction error test (Fig 4). The mean surface error was less than 0.2 mm after the inclusion of the first 5 PCs of variation and less than 0.1 mm after the first 13. These values equate to approximately 0.7% and 0.35% of the mean canine tooth length respectively.

**Fig 5:**
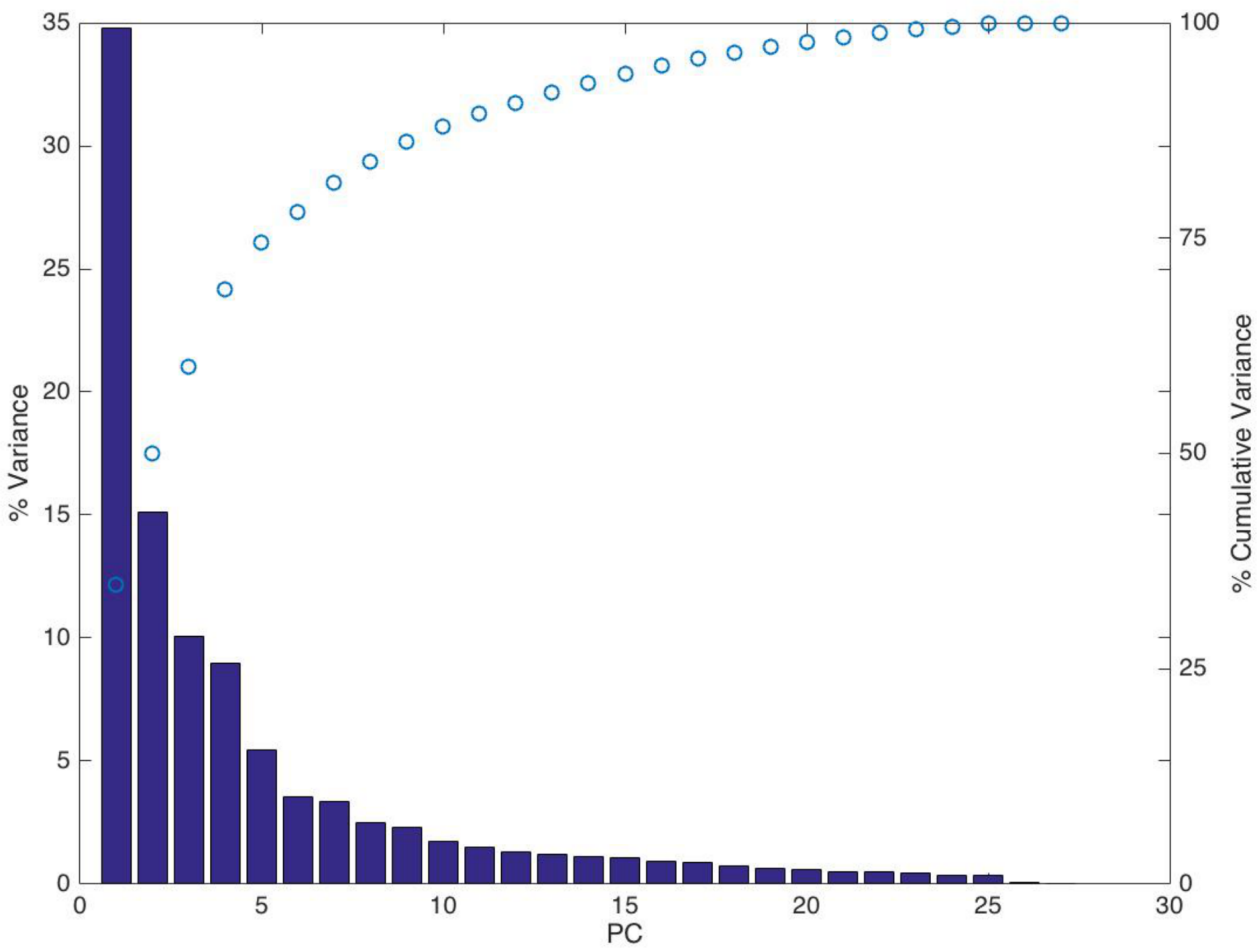
SSM variance. % Variance (bar) and cumulative variance (circles) captured by PCs.

The variance contained within each PC was calculated using the covariance matrix. Ranked from highest to lowest, this produced a decaying exponential variance plot (Fig 5), typical of past PCA studies [55], with the first PC capturing over 30% of the total variation. By the 5^th^ PC approximately 75% of the variance was captured, so the first five PCs were chosen for subsequent analysis.

### Automated measurement

**Fig 6:**
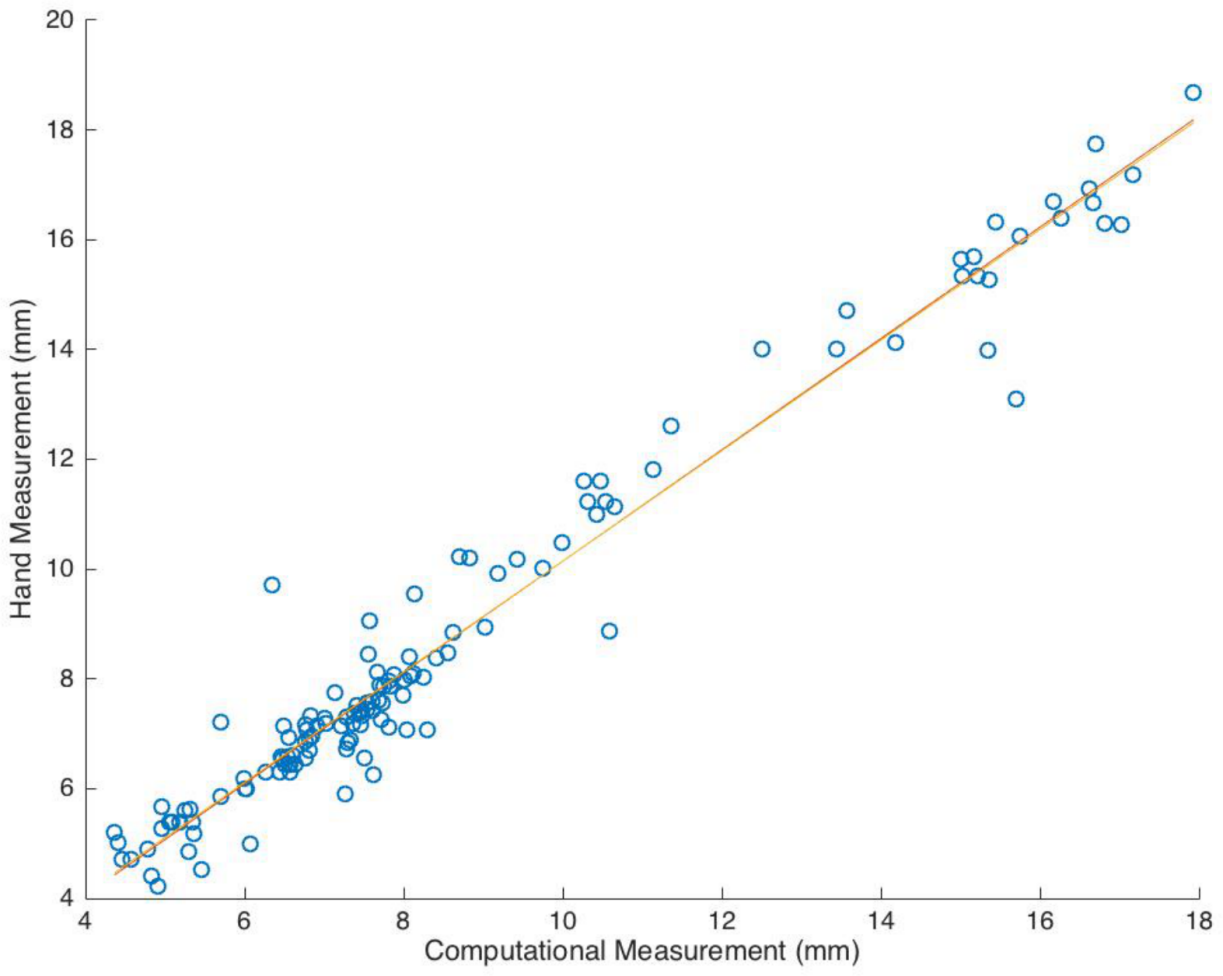
Measurement regression. Combined regression for all 6 probed anatomic measurements on 27 training samples.

**Fig 7:**
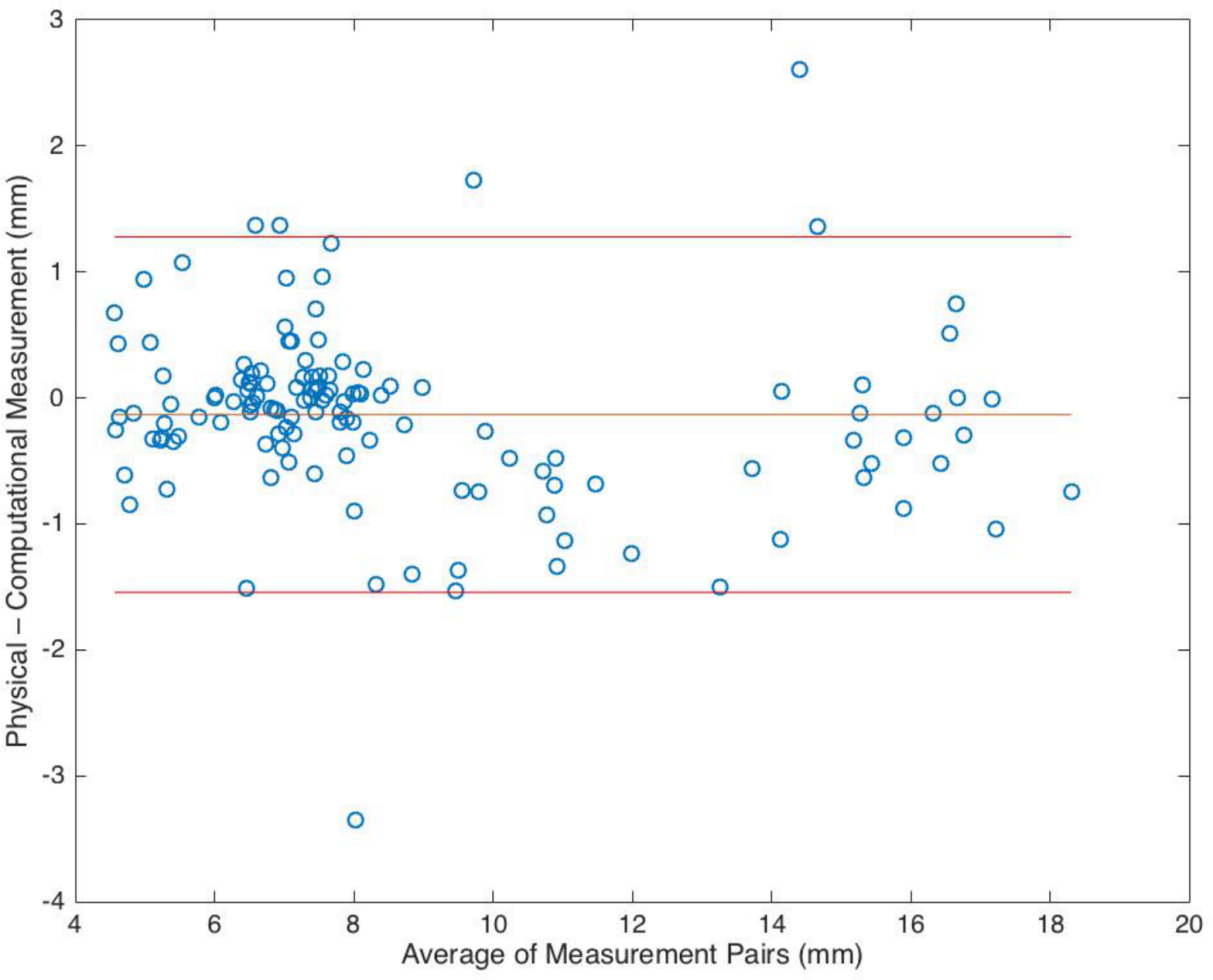
Bland-Altman. Bland-Altman plot for all 6 probed anatomic measurements on 27 training samples. Mean difference and 95% limits of agreement indicated.

Comparison of the automated smoothing, landmark assignment and measurement routine with manual calliper measurements showed a regression (R^2^) of 0.96 and a gradient of 1.014 (Fig 6). Bland-Altman plots revealed independence of pairwise measurement differences from measurement size (Fig 7). The mean error was -0.13 mm between the mean automated and hand measurements, and the 95% confidence interval was from -1.54 to 1.28 mm.

### Principal component analysis

#### Mode shapes

**Fig 8:**
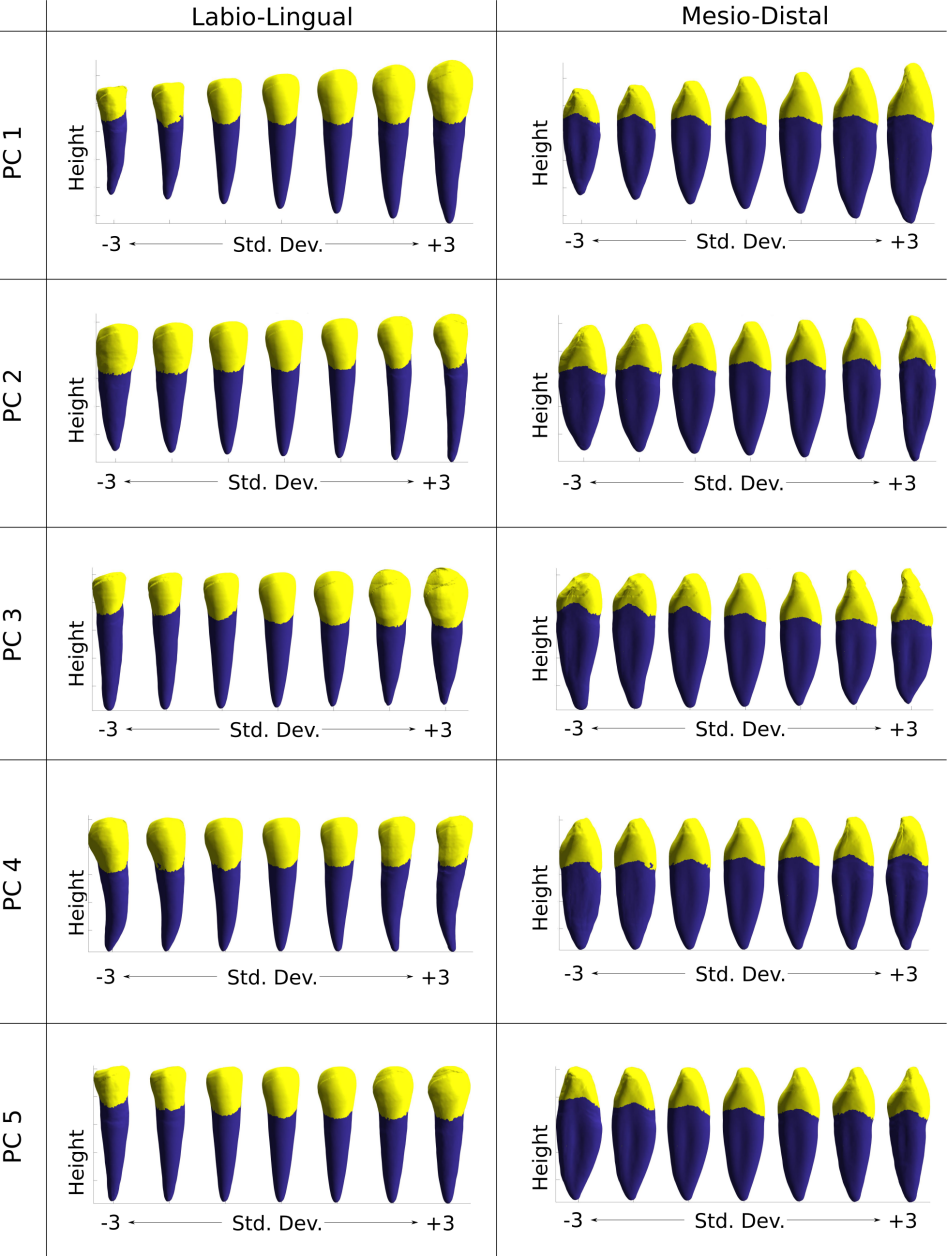
PC visualisation. Plots of physical attributes associated with manipulation of the weighting coefficient (+/-3σ) of each PC shape.

The predominant attribute observed in the first PC was overall scaling (Fig 8A). This observation was supported by the dimensional data that showed an increase in all probed measurements across the range. A significant correlation was identified between PC 1 and all of the probed measurements—more than for any of the other PCs (Table 3, PC 1 in S3 Fig). The change in overall scale was most apparent in the volumetric data that indicated a change in volume of >90% across +/–3σ. Other morphological changes included mesio-distal root curvature and crown flattening. These secondary characteristics were harder to identify using measurements as the effect of overall scaling dominated the data.

**Table 3:** Regression (R^2^) analysis of probed measurements against first 5 PCs for +/-3 standard deviations.

PC	LOCla	LORla	MDC	MDCc	LDC	LDCc	Area	Volume
1	0.978	0.973	0.987	0.977	1	0.867	0.995	0.992
2	0.411	0.992	0.987	0.976	0.996	0.945	0.995	1
3	0.985	0.971	0.955	0.63	0.674	0.002	0.039	0.981
4	0.702	0.94	0.917	0.687	0.989	0.515	0.536	0.952
5	0.93	0.838	0.868	0.776	0.882	0.179	0.812	0.982

Significant (p-value < 0.01) linear regressions shaded grey.

PC 2 was associated with two main characteristics: mesio-distal and labio-lingual root widths, as identified by the MDCc and LDCc measurements respectively. Both these measurements were significantly correlated to PC 2 (Table 3, PC 2 in S3 Fig) and were indicative of the observed increased root diameter, particularly around the CEJ (Fig 8B). These changes were reflected by the high correlation with tooth volume. The crown geometry was less affected by PC 2 weighting.

Conversely, PC 3 exhibited high correlation with crown length (Table 3, PC 3 in S3 Fig) and, to a lesser extent, root length and crown diameter. Inspection of the shape variation (Fig 8C) indicated changes in root tip taper, a feature not identified by the computed measurements.

Quantitatively, the results suggest that PC 4 had less influence on the probed measurements (Table 3, PC 4 in S3 Fig). However, observation of the physical PC plots identified the expression of root curvature, symmetrically ranging from the mesial to distal aspect (Fig 8D). Similarly, PC 5 had limited influence on the quantitative measurements of the tooth compared with other PCs. Crown and root length were the most affected characteristics (Table 3, PC 5 in S3 Fig); crown-root ratio was the most defined attribute, as a reduction in crown length was associated with an increase in root length.

### Sample characterisation

**Fig 9:**
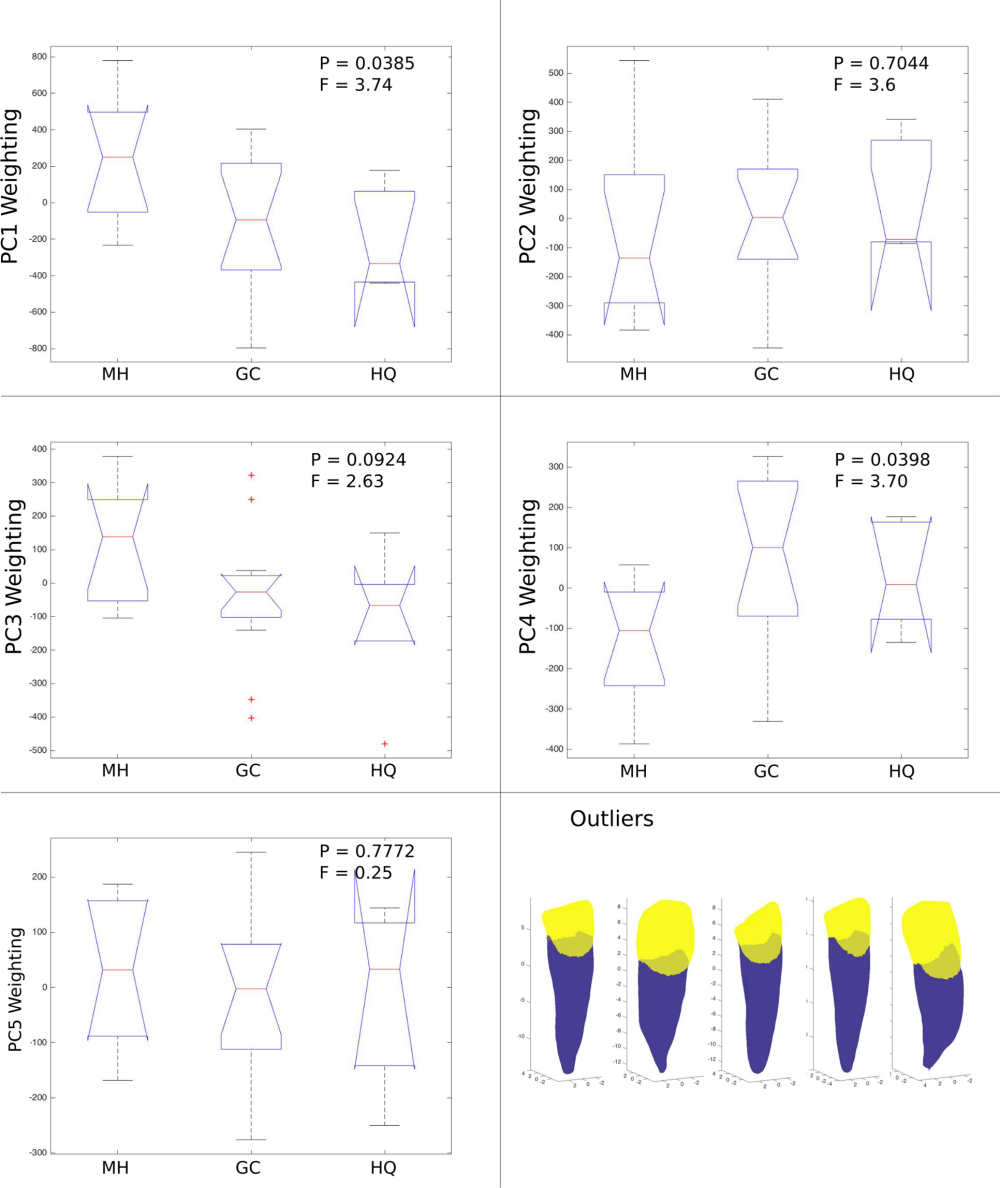
Sample boxplots. Box plots of PC weightings for samples. Boxes showing 25^th^ and 75^th^ percentiles, whiskers extend to data extremes (not including outliers), red crosses represent outliers and notches illustrate medians with 5% significance indicated if their intervals (extremes of notches) do not overlap. Principal component weighting coefficient F-statistic (ratio of mean squares) and P-values for samples in one-way ANOVA test. Outliers identified in PC 3 plotted at bottom right.

The samples were compared using boxplots of the PC weighting coefficients (Fig 9). Statistically significant differences (P<0.05) between samples were seen in the first (MH and HQ) and fourth (MH and GC) PCs. There was little difference in the coefficients for PC 2. PC 3 exhibited higher weighting for the MH sample than GC and HQ, but this was not significant. PC 3 indicated 5 outlier data points, which were observed to be instances of extreme geometric attributes, i.e. either small crowns lengths (bruxed) or highly tapered and curved roots. No trends were observed in the 5^th^ PC.

## Discussion

This study presented SSM as a supplementary principal components method for GM analysis of human teeth. Geometric and material information from CT was used to build a SSM to identify the dominant PCs within the samples. A material approach was included to distinguish between dentin/cementum and enamel on the tooth surface to enable identification of the cemento-enamel junction. The derived material and geometric information was used to implement an automated landmark identification algorithm that measured the sample geometry. The resulting models were used to demonstrate how researchers might identify dominant morphological variations within a sample and between sample groups.

### Standard measurements

The samples were representative of modern populations, by comparison to reported average mandibular canine measurements [10, 69]. The process of automated landmark measurement was validated against hand calliper measurements using regression (Fig 6) and Bland-Altman (Fig 7) analysis and previously validated for intra-observer error (cervical error measurement 0.3–0.5% (S1 Text)). The analysis of the two methods identified a strong correlation that was independent of measurement size. Variations in measurement were predominantly attributed to differences in landmark locations (post CEJ smoothing) between the two methods. However, these differences were small as a proportion of the gross crown and root dimensions, and are not thought to affect the validity of the current study, as samples were compared using non-dimensional trends corresponding to principal components.

### 3D measurement

Prior studies have been conducted using CT to investigate the mineral content/composition of teeth [70–74]. The current study used mineral density to identify enamel and cementum/dentin material types, infer the CEJ profile and statistically model its variation. It was found that the CEJ profile varied with PC shape and weighting, and provided further evidence of the differences in root/crown morphology and their interactions. Others have found similar material variations in femoral specimens using statistical shape and intensity models (SSIM) [55, 75], which capture the dominant variations in volumetric material distribution that exist alongside the geometric variations.

The current study’s micro-CT and computational measurement approach also enabled investigation of surface areas and volumes. Strong correlations were observed between both these measures and the first and second PCs. Micro–CT has been used to make basic linear measurements of internal tooth attributes [76], and advanced measurements such as whole-tooth and pulp chamber volumes and morphologies [77–79] for the purpose of aging individuals. Volumetric measurements using micro-CT have been validated using water displacement and have found agreement between -4% and 7% [80].

### 3D morphometrics

Although GM analysis has been applied extensively within dental archaeology and anthropology [27–45, 81, 82], most of these studies have used either outline or surface methods and have focused on the occlusal surface of the crown. To date, only two GM studies have analysed root variation [42, 46] and no studies have been studied whole tooth shape variation. Only a limited number of studies have handled volumetric data, despite morphometric maps having been used to successfully demonstrate fine levels of variation and subtle characteristics of root architecture [83, 84].

To our knowledge, Fernee and colleagues [46] provide the only instance of the application of GM to teeth in an archaeological comparison of historical and modern samples, and the present study uses GM, by virtue of SSM, to give a fuller picture of tooth size and shape variation. Linear, area and volumetric measurements were used to capture size attributes quantitatively, whilst shape attributes were captured using full field geometric and material plots with respect to principal component variation. This method of combined size and shape representation provides a platform for new dental measurements, which is currently being pursued in the field of dental phenomics [85, 86].

### Mode analysis

#### Size

The primary variation observed in the statistical model was overall size, associated with PC 1, which accounted for 35% of the total sample variance. This finding is consistent with existing studies of biological structures using geometric principal components. In femurs, it has been previously reported that, using a statistical shape and intensity approach, PC 1 corresponds with size and is responsible for between 30% and 45% variation [55, 75, 87]. In teeth, it has been reported that approximately 48% of the variability is captured by the PC 1 in a premolar model, which again strongly corresponds with total tooth size [88]. In the same study, it has been suggested that physical attributes other than total size are present in the PC 1. This is true of the data presented in the current study, whereby crown wear and, to a lesser extent, root curvature are visible in the first PC1.

Linear mesiodistal and bucciolingual crown diameters have been investigated using a PCA approach to determine differences in geographic populations, and PC 1 was found to represent overall size [6]. Similarly, tooth size measurements, both with and without mechanisms to avoid multicollinearity, have been recently noted as being of particular interest for assigning geographic (and hence potentially genetic) ancestry [89]. In this study, PC 1 correlated well with all linear, area and volumetric measurements, so a consensus can be drawn that expression of PC 1 could be a suitable metric for sample size comparison.

#### Crown wear

As an accepted function of age in the past, a number of qualitative [58, 90–94] and quantitative [94–98] methods have been developed to measure tooth wear. Few of these methods offer a protocol for assessing wear in anterior teeth, but instead offer either an observation or a measurement.

Crowns displaying a moderate degree of wear (Molnar < 6) were included in the training dataset for the statistical model, enabling investigation of crown wear and its links with morphology. Crown wear was identified in the 1^st^ and 3^rd^ PCs. Particularly in PC 1, wear was associated with a negative weighting, and corresponded with (or was confounded with) small size (Fig 8). Analysis of weightings between samples (Fig 9) indicated that the archaeological samples would be expected to have lower weightings, and thus a trend can be observed with archaeological samples being typically smaller and demonstrating greater extents of wear. PC 3 may be indicative of a functional link between root size and extent of wear, as larger roots are more stable and can withstand greater forces without incurring dentoalveolar injury [99, 100]. Although tooth wear is likely an indicator of vigorous masticatory activity, it is not necessarily correlated with the nature of the force. However, tooth wear is often used as a proxy for loading and thus diet [42]

It is an interesting observation that crown wear could be quantitatively explained using the degree of expression of a particular PC (or combination of PCs) that describe a registered tooth. However, it must be noted that the current model was not optimised for crown wear identification; indeed, a specific contraindication for inclusion was advanced wear. It is feasible that a model containing a broader spectrum of worn teeth would give a more positive identification and characterisation of crown wear. However, achieving good nodal correspondence during registration would be difficult, therefore registered mesh quality metrics must be observed. Also, care must be taken not to bias the model to any particular trait, and random sampling would be preferred.

#### Root form

The majority of tooth root studies provide qualitative reports, with an absence of metric analysis [101, 102], or with measurements limited to height [101]. This study has included a number of measurements which provide an insight into changes in root form. It was seen that in PC 1 the root length was related to crown length as an attribute of overall size, PC 2 contained variation in root slenderness, and in PC 3 root length was inversely related to crown length. As previously mentioned, this inverse relationship proved to be another useful indicator of tooth wear, and outliers (Fig 9) were all found to have higher levels of crown wear and/or abnormal crown-root ratios [69].

Non-metric visualisation and comparison of the PCs allowed for analysis of attributes that would be otherwise overlooked using traditional linear measurements. Root apex acuity was one such attribute suggested by the variation visualised in PC 3, whilst root curvature was the most dominant feature of PC 4. With knowledge of these attributes from the qualitative statistical analysis it is possible either to devise new measurement protocols, or to use the PC weighting coefficient itself to describe the expression of the attribute quantitatively, as this is a direct description of the morphology in three dimensions. Identification of such traits demonstrates the advantage of SSM analysis, as the model identifies the dominant shape attributes in a sample so that the observer can be strategic in designing measurement methodologies to capture them.

#### Pathologies

It is possible to detect and characterise pathologies using anatomical SSMs. Femoroacetabular impingement morphology has been investigated using statistical shape models of femurs [103], whilst landmark based SSMs have been used to detect differences in the paediatric airway of subjects with and without cystic fibrosis [104]. SSM has also been successfully used to discriminate between patient groups expected to develop osteoarthritis by evaluating bone surface geometry of the knee joint [105]. The current study did not set out to identify pathologies, but minor abnormalities were identified on the root in the region of the CEJ in the extremes of the third and fourth modes of variation. This concavity beneath the CEJ was akin to damage or an abfraction lesion.

### SSM, GM and their archaeological potential

The past 25 years have seen fundamental changes in the field of morphometrics, with a shift in how morphology is quantified through the use of GM [19]. Quantification methods have been developed from the introduction of the use of landmark co-ordinates towards the use of semi-landmarks to analyse outlines and surfaces. GM is capable of overcoming some of the caveats of non-metric traits and metric measurement analysis. It overcomes the potential subjectivity of non-metric traits [7], and detects the shape information lost by metric measurements. SSM is akin to an exclusively semi-landmark based approach, with a semi-landmark on every point of an object’s surface, up to the spatial resolution of the scanning method. By using SSM the need to infer or interpolate information between landmarks and/or semi-landmarks is removed, thus resolving more of an object’s surface. This study demonstrates how subtleties such as root curvature can be clearly identified by fully resolved surface models, which may have been more difficult to identify using conventional GM methods. SSM is also valuable in instances when analysing forms deficient in absolute landmark points, and may be more time-efficient as the process is fully automated.

The current study highlights the advantages of using a material capturing SSM. In this instance, material information was recoded through the use of CT grayscale values, but it would be feasible to achieve similar results using white light surface scanners, providing the change in material was demarked by a colour gradient. Here, it was seen how material identification could be used to automatically identify the CEJ, thus enabling the analysis of established odontometric measures. It is a small step to envisage how material distinctions in GM could be used in other applications, for example the transition between cortical and trabecular bone, the interface between parts of tool, or indeed any object comprising of two or more materials.

Dental studies of geographical variation have been used to study biodistance, to investigate population history, migration and kinship patterns [106–108]. Only one study is known to provide GM analysis of geographical variation, estimating biological affinities [29]. Metric and non-metric traits have been combined to study migration and biological affinity, and the results evaluated in comparison with Sr isotope results [108]. GM can provide quantitative assessment of shape thereby highlighting the possibility that these analyses of teeth may be used in future as a non-destructive tool to detect migratory patterns and biological affinity. GM has been used to address questions of hominin evolution and taxonomic variation. Future research in hominin evolution and taxonomic variation would benefit from the application of new methods, such as SSM. These may be of particular importance in the taxonomic identification fossil teeth that are often discovered in isolation [35, 42].

Using SSM and GM, it may be possible to develop microevolutionary, environmental, social and biological models, like those produced from hominin dental samples [42, 109, 110]. This is supported by Fernee and colleagues [46], where the potential of the application of GM surface analysis of tooth roots in the production of dietary models is illustrated. Variation within these samples could also be analysed on an individual level. This includes the analysis of morphological sexual dimorphism in the dentition that has already been studied using linear measurements [111–115].

Forensic odontology also can benefit greatly from the application of SSM and GM. Dental information can inform all aspects of biological profiles, including age, sex and ancestry [103]. This could enable age estimation through the identification and analysis of the morphology of degenerative changes, such as tooth crown wear and signals of stress reflected in root and root chamber morphology [1, 78, 79, 116, 117].

### Limitations

A limitation of the current study is the size of the training dataset. In total, the sample size used to compile the model is similar to SSMs built by others [51, 55, 88, 103–105, 118]. However, the individual sample sizes were limited. This may be one reason why distinct trends were difficult to identify in the PC comparison. With a larger dataset it would be possible to construct independent statistical models for each sample group [103, 104]. This differing strategy could highlight major differences in the samples by comparing the differences in the PCs.

Canines were selected as the most stable tooth within the dentition, according to Butler’s field theory [56]. This may have affected the degree of variation that was detected within and between samples. In future work, it may be of interest to consider teeth which exhibit greater variability, such as posterior teeth.

Identification of the CEJ is essential for understanding the geometry of the tooth and the relationship between the root and crown. The synthetic outputs from the SSM were binarised in order to identify a finite CEJ. This binarisation was coupled with a smoothing and isolated material removal routine to give an anatomically feasible result. However, this smoothing process may affect the derived CEJ measurements. An alternative to smoothing the CEJ would be to build a multi-body SSM consisting of separate crown and root meshes. Similar strategies have been demonstrated for the knee joint complex using either integrated mesh-morphing segmentation algorithms [119] or multi-body alignment models from magnetic resonance images [118].

### Potential exploitation

SSM methods, as exemplified in the current study, have a large potential for use alongside GM in the identification of osteological samples [52]. GM with SSM could improve on existing metric and non-metric dental methods, for forming age, sex and ancestry profiles, and for example enable age estimation through analysis of tooth crown wear and other detailed morphological features [1, 78, 79, 116, 117].

Considering information loss, it has been demonstrated how dental anatomy missing as a result of wear may be predicted using SSM [88]. This is achieved by comparing a partial (i.e. worn) shape to the model and finding optimal coefficient weightings to fit the partial shape to the SSM. Forensic odontology, and a host of other (non-dental) disciplines, could exploit this method to digitally reconstruct samples with partial or damaged material.

The presented data are a subset of a larger model which the authors have collected, representing all single-root anterior and premolar teeth. Such datasets have the potential for extensive osteoarchaeologic, forensic and biomedical engineering use. This includes the development and pre-clinical analysis of dental prostheses, and extensive multi-individual computational modelling (FEA) examining population-wide biomechanics.

Given the fragmentary nature of the archaeological record, using SSM has potential to aid in archaeological reconstruction of artefacts or collections. This paper has demonstrated that, by synthesising material properties with shape, additional information may be extracted from artefacts. Although the current focus has been on osteoarchaeology, SSM may be beneficial to most archaeological analysis, such as of inlaid ceramic sherds and vessels, thereby adding nuance to understandings of object biographies including provenance, trade patterns, exchange networks, use and discard.

## Supporting information

S1 Fig Alignment GUI. (PDF)

S2 Fig CEJ smoothing procedure. A) smoothing routine if two bordering elements of different types are identified. B) smoothing routine if three bordering elements of different types are identified.
(PDF)

S3 Fig PC regressions. Percentage change in measurements from mean geometry across +/-3σ of weighting coefficient for each PC (1–5) (left), and corresponding regression plots (right).
(PDF)

S1 Text Intra-observer error. (PDF)
