# Supplementary figures and images for: The potential of statistical shape modelling for geometric morphometric analysis of human teeth in archaeological research
Source: PLoS One. 2017 Dec 7;12(12):e0186754. doi: 10.1371/journal.pone.0186754 (PMC5720725; doi:10.1371/journal.pone.0186754)

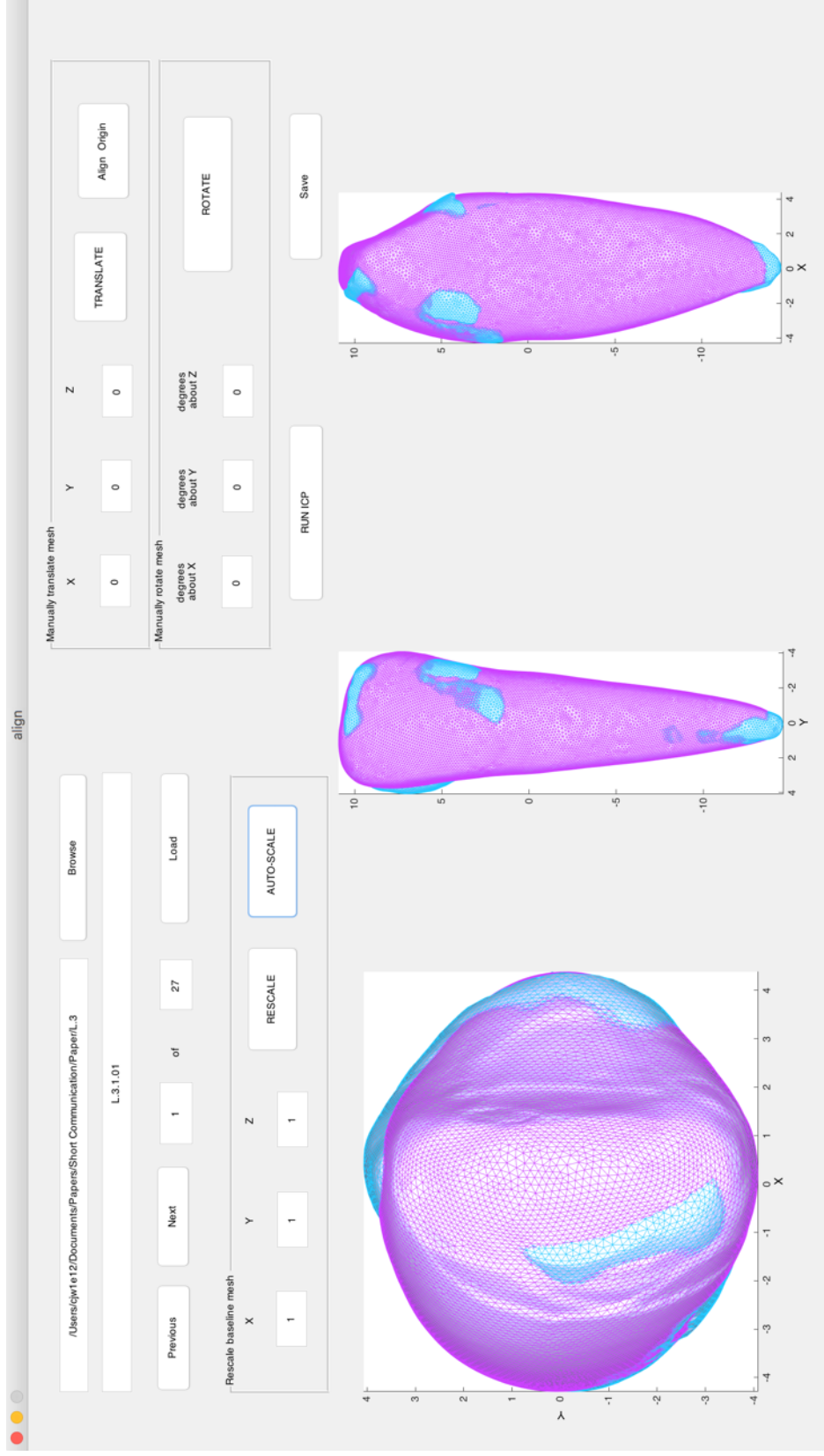

Supplement: S1 Fig — (PDF) [file pone.0186754.s001.pdf]

## CEJ Smoothing

A. Two isolated element edges

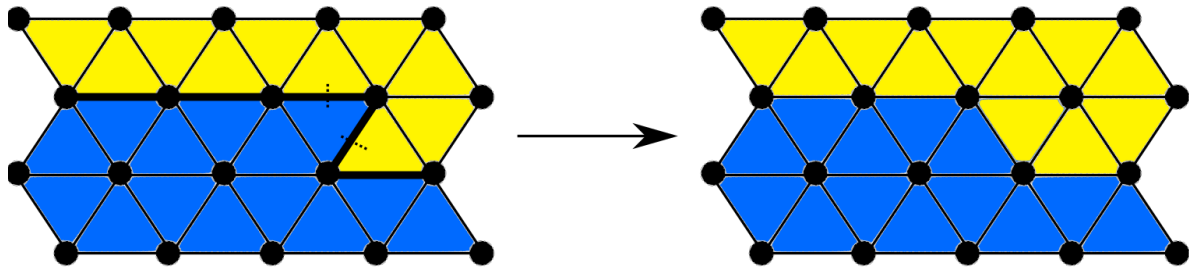

B. 3 Isolated element edges

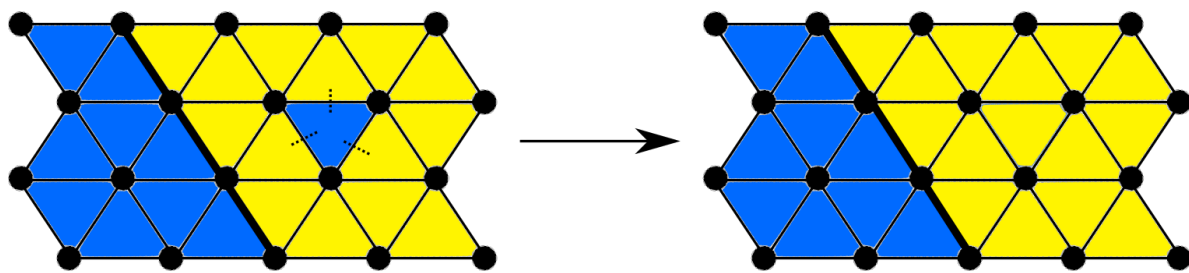

Supplement: S2 Fig — A) smoothing routine if two bordering elements of different types are identified. B) smoothing routine if three bordering elements of different types are identified. (PDF) [file pone.0186754.s002.pdf]

## PC1

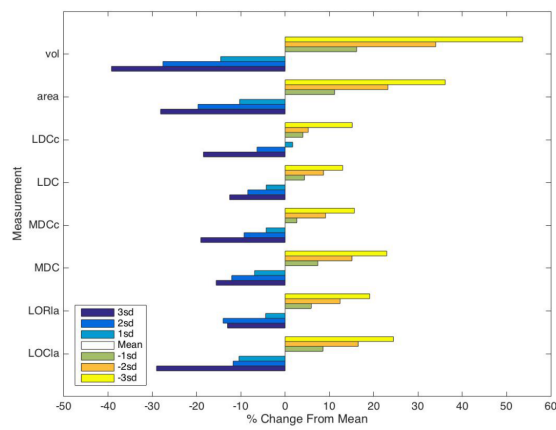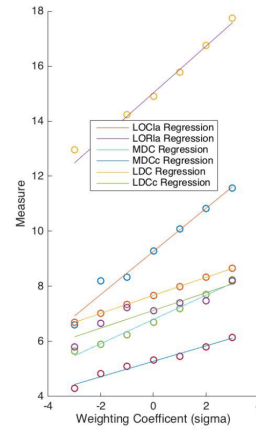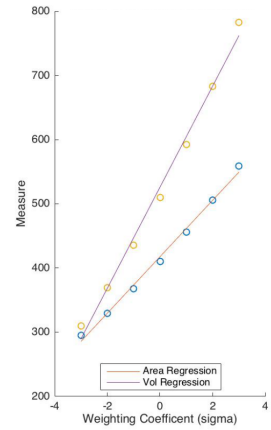

## PC2

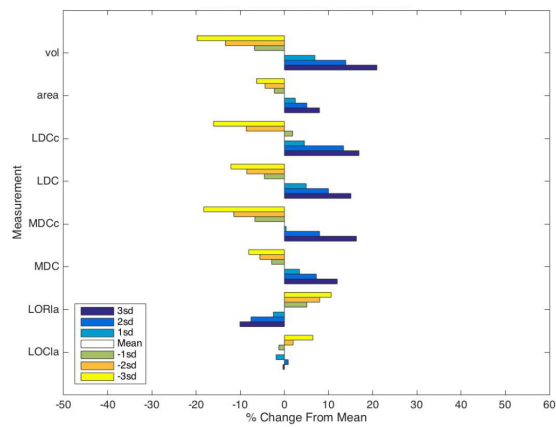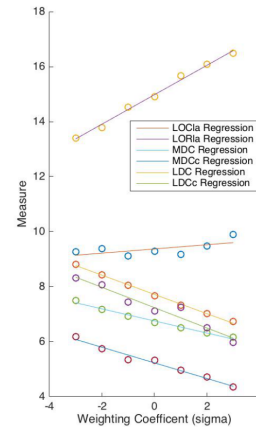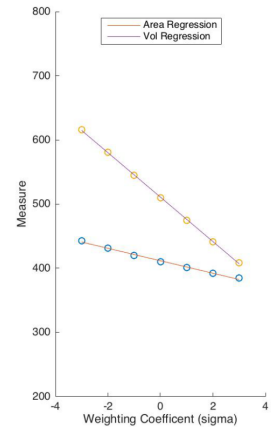

## PC3

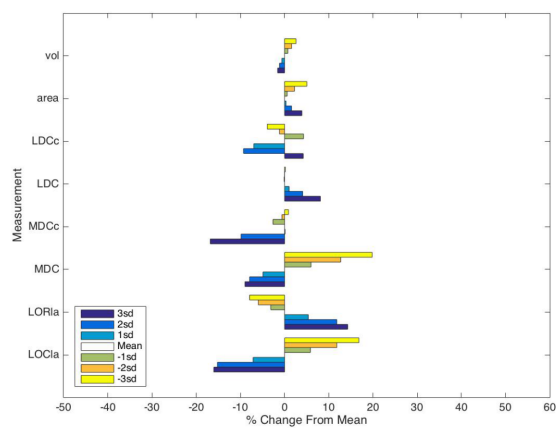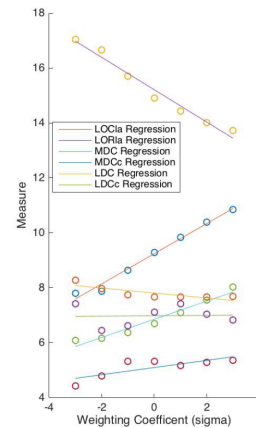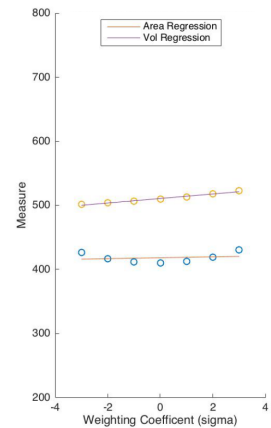

## PC4

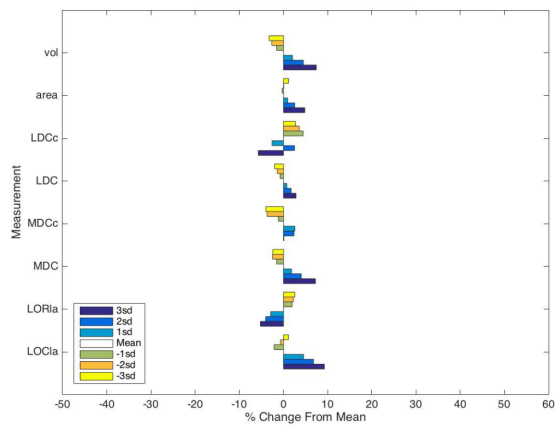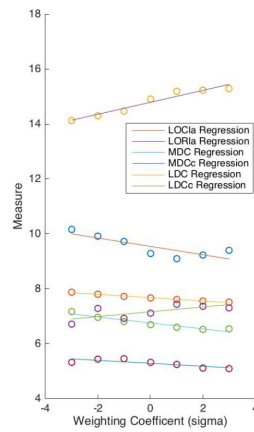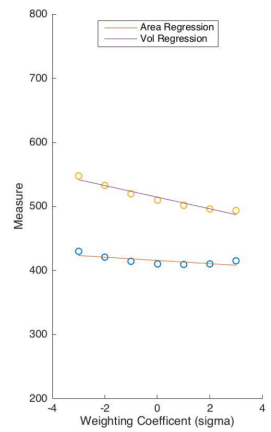

## PC5

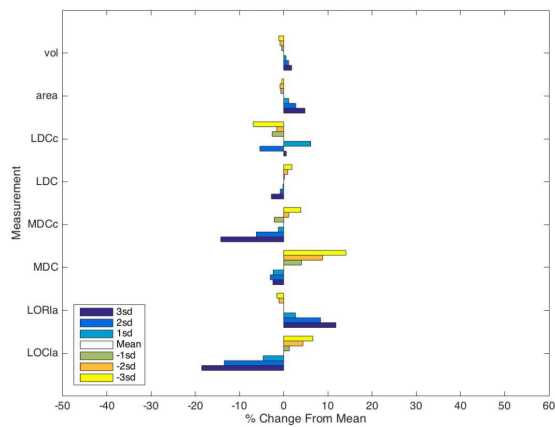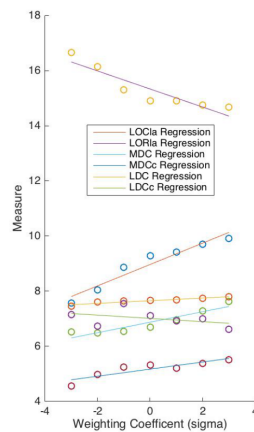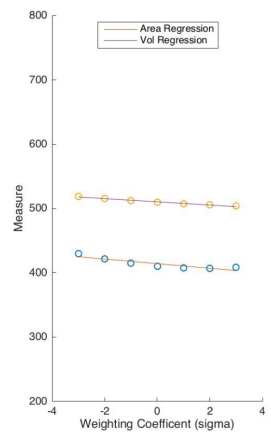

Supplement: S3 Fig — Percentage change in measurements from mean geometry across +/-3σ of weighting coefficient for each PC (1–5) (left), and corresponding regression plots (right). (PDF) [file pone.0186754.s003.pdf]
