# Supplementary material for: The potential of statistical shape modelling for geometric morphometric analysis of human teeth in archaeological research
Source: PLoS One. 2017 Dec 7;12(12):e0186754. doi: 10.1371/journal.pone.0186754 (PMC5720725; doi:10.1371/journal.pone.0186754)
Supplement: S1 Text — (PDF) [file pone.0186754.s004.pdf]

Intra-observer Error

3.2.4.2 Intra- Observer Error

The manual CEJ measurements were tested for precision and reproducibility. This attempts to quantify the inherent precision of single observation by repeating it, assuming that the replicate difference are normally distributed (Kieser 1990:14). 10 archaeological and 10 modern teeth wear re-measured, selected to cover all tooth types and ages and both sexes in archaeological samples. The results were analysed using the method error statistic. The results for these can be found in Appendix 2.1.

Appendix 2.1 Error statistic

Standard deviation was calculated. Then the technical error measurement (TM) ($\sqrt{\sum d^2 / 2n}$) was calculated, where d is the difference between repeated measurements and n is the number of repeat measurements. Then the contribution of measurement error to sample variance was calculated (TM^2/SD^2). This was done for both the mesiodistal and the labiolingual diameter of the cervix.

Mesiodistal diameter at the cervix = 0.0047 (0.5%)

Labiolingual diameter at the cervix = 0.0029 (0.3%)
